# Myasthenia gravis and nutrition

**DOI:** 10.3389/fnut.2025.1600508

**Published:** 2025-07-09

**Authors:** Merve Nur Uçak, Gözde Arıtıcı Çolak

**Affiliations:** Department of Nutrition and Dietetics, Institute of Health Sciences, Acibadem Mehmet Ali Aydinlar University, Istanbul, Türkiye

**Keywords:** myasthenia gravis, nutrition, diet, inflammation, microbiota

## Abstract

Myasthenia gravis is an autoimmune neurological disease characterized by a disorder of nerve transmission at the neuromuscular junction. In this disease, antibodies attack acetylcholine receptors and their associated molecules, disrupting nerve transmission and impairing normal muscle contraction. Myasthenia gravis is diagnosed through physiological and neurological examinations, blood antibody tests, and electronic methods. Various approaches are used for symptom management, including immunosuppressive drugs, acetylcholinesterase inhibitors, and thymectomy. Previous literature has focused primarily on symptoms and medical treatment. However, resources related to nutrition and Myasthenia gravis are limited. This study bridges this gap by examining the effects of different nutritional interventions on the disease and the importance of nutrition in the treatment process.

## 1 Introduction

Myasthenia gravis (MG) is an autoimmune disease in which antibodies attack the acetylcholine receptors and their associated molecules (e.g., muscle-specific kinase and lipoprotein receptor-related peptide 4) on the postsynaptic membrane at the neuromuscular junction. These antibodies prevent acetylcholine from binding to receptors, causing problems in nerve transmission. This also causes weakness in the skeletal muscles, either generally or localized, and this muscle weakness worsens with repeated use of the muscles (1). Because this issue with nerve transmission affects the muscles in the face, throat, and diaphragm, individuals may experience weakness in the eye muscles, ptosis, and diplopia. They may also experience difficulties with speech, shortness of breath, and trouble swallowing, which directly impacts feeding (2).

The MG is caused by a complex gene–environment interaction. In the diagnosis process, physical and neurological examinations, electrophysiological methods like repetitive nerve stimulation, and blood tests evaluating antibodies can be used. Because weakness is a common symptom of many other disorders, it can be challenging to diagnose MG in individuals experiencing mild weakness or weakness limited to only a few muscles (2, 3). Although the prevalence of the disease varies geographically, cases have increased worldwide over the past 50 years, with a prevalence of 150–200 cases per million people (4). It is most commonly diagnosed in women under 40 and men over 60 (2).

The etiological process in which autoimmunity occurs causes the development of an inflammatory response. The immunological interactions between the autoantibodies of the neuromuscular junction and T helper cells lead to the activation of proinflammatory cytokines like TNF, IFN-y, and IL-17. The abnormal and irregular activation of regulatory T cells also plays a significant role in the inflammatory process and suppressing the immune system (5).

### 1.1 Myasthenia gravis and autoimmune comorbidity

Multiple autoimmune diseases tend to be observed together due to the scope of immune system disorders. Studies have reported the comorbidity of MG with celiac disease (6–8), thyroid disease (9), and multiple sclerosis (10).

Celiac disease is a chronic autoimmune disorder of the small intestine triggered by gluten consumption in genetically predisposed individuals. Gluten intake causes inflammation in the intestinal mucosa and villous atrophy, leading to the malabsorption of nutrients (11). A gluten-free diet is implemented for its treatment. In one case of comorbidity, a patient with gastrointestinal symptoms, fatigue, and muscle weakness complaints was diagnosed with both celiac disease (through histopathological examination and serological tests) and MG due to neuromuscular symptoms and positive acetylcholine receptor antibodies (7). As treatment, a gluten-free diet was applied for the celiac disease, and immunosuppressive therapies were used for MG. It has been noted that the connections between gut health and autoimmune mechanisms may play an important role in managing comorbid celiac disease and MG (6). However, in a large-scale cohort study, it was noted that the relationship between the diseases was not as pronounced in the general population as in case studies. It was therefore concluded that celiac disease does not increase the risk of MG and that there is no significant relationship between them (8).

Meanwhile, autoimmune thyroid diseases are more common in patients with MG compared with the general population (9). Hashimoto’s thyroiditis and Graves’ disease are the most common thyroid disorders in patients with MG. Fluctuations in thyroid functions can exacerbate MG symptoms, and immunosuppressive methods may be required for treatment. It is therefore recommended that the thyroid functions of patients with MG be monitored regularly (9).

Finally, it has been reported that multiple sclerosis, characterized by demyelination in the central nervous system, can rarely be seen together with other conditions. In the pathogenesis of both diseases, dysfunctions in T and B cell functions play a role. To develop more effective treatments, it is necessary to investigate the relationship between the mechanisms involved (10).

### 1.2 Gaps in myasthenia gravis treatment

Although there is no known cure for MG, methods have been developed methods to reduce or slow muscle weakness. The “International Myasthenia Gravis Management Guide” (12) details the methods and mechanisms used in the treatment of the disease. The first step in treatment can include acetylcholinesterase drugs, immunosuppressants, plasma exchanges in acute increments, or thymectomy in patients with thymoma.

However, there is little information about nutrition in mainstream guidelines. Indeed, the impact of dietary changes on neuromuscular diseases is understudied, though positive relationships have been reported (13). Of the few guidelines that touch on nutrition and MG, information published by the American Myasthenia Gravis foundation, the dietary recommendations emphasize a balanced and healthy diet of small, frequent meals whose contents should be determined by age, gender, weight, and exercise level. The authors recommend having meals during periods of high energy to limit the symptoms and include advice regarding medication, food consistency, and eating position (14). However, this guidance is over 30 years old and in significant need of update. Only good nutrition is emphasized among the lifestyle changes that will increase patients’ overall self-efficacy (15).

Current work on MG and nutrition is extremely limited. Studies on this field are especially lacking in the worldwide. For example, a brief section of the booklet titled “Information about Myasthenia Gravis for Patients and Their Families” by the Turkish Neurological Society’s Scientific Study Group on Neuromuscular Diseases, it is stated that no special diet is recommended for the disease and that patients merely need to maintain a balanced diet (16). The aim of this study was to fill this gap by investigating the effects of different nutritional interventions on MG and to demonstrate the importance of nutrition in treatment.

### 1.3 Methodology

This review aims to evaluate the effect of different dietary interventions in MG patients and to determine the most appropriate dietary model for this group. The compilation question was determined as follows:

- Do different dietary interventions have an impact on disease-related symptoms or disease prognosis in individuals with MG?

The search was carried out between January 2025 and March 2025 in three different databases: ‘PubMed’, ‘Web of Science’, and ‘The Cochrane Library’ over the university’s access network. Searching in these databases was carried out using [(intermittent fasting) OR (plant based diet) OR (vegetarian diet) OR (vegan diet) OR (ketogenic diet) OR (Mediterranean Diet) OR (gluten free diet) OR (vitamin supplementation) OR (mineral supplementation) OR (probiotic) OR (dietary interventions)] AND (myasthenia gravis) keywords. Review, meta-analyses, case reports, clinical study, clinical trial, observational study, randomized controlled trials were included. For research studies, only those published within the last 10 years (17) were considered for inclusion.

## 2 Nutrition and myasthenia gravis

Myasthenia Gravis is a neuromuscular disorder that can impact various aspects of a patient’s health, including nutritional status. Nutrition issues are common for patients with MG, potentially due to the difficulties the disease causes with swallowing (dysphagia). It has been reported that dysphagia is observed in approximately two-thirds of individuals with MG due to the fatigue of the muscles involved in chewing and swallowing (18, 19). As a result, loss of appetite, malnutrition, and an overall decline in patients’ quality of life (QoL) are frequently observed (20). In a case study of an 86-year-old woman with MG, it was reported that she was unable to eat adequately due to dysphagia and had experienced significant weight loss (21). A study of 104 adults with MG found that individuals with a poor disease course had higher rates of dysphagia and hypoalbuminemia. Therefore, coordinated multidisciplinary care involving dietitians, neurologists, and speech therapists is essential for the effective management of dysphagia and nutritional status in patients with MG (22).

In another study, a 70-year-old patient with dysphagia who experienced involuntary weight loss over 2 years was given magnesium intravenously as part of another treatment process. It was observed that magnesium increased the neuromuscular blockade and significantly worsened the patient’s symptoms. After the infusion, the diagnosis of MG was made on the basis of the worsening of muscle weakness and dysphagia, along with antibody and electrophysiological tests (23). This demonstrates that MG can manifest solely as dysphagia and that neuromuscular causes should therefore be considered when evaluating dysphagia, especially among the elderly.

Malnutrition, primarily due to dysphagia, has been associated with higher Controlling Nutritional Status (CONUT) scores in patient with MG (24). As dysphagia may contribute to nutritional deficiencies and malnutrition, monitoring this symptom is crucial for preserving or improving patients’ nutritional status and enhancing their QoL. Malnutrition has also been associated with an increased risk of dyspnea (25), a common issue for patients with MG. It should be noted that while malnutrition and higher CONUT scores have been reported in MG patients, this reflects an association rather than a proven causal relationship.

Considering that patients with MG are at risk of malnutrition, ensuring adequate, balanced, and appropriate nutrition is of great importance. Dysphagia is often caused by fatigue; therefore, it is important to provide nutrient-rich foods at the beginning of a meal. Meals should be small, frequent, and contain foods that are relatively easy to chew and swallow. Additionally, exercise should not be performed before meals (26). Additionally, it is important to routinely screen the nutritional status of patients with MG to determine malnutrition and provide a diet that meets their current needs (25).

Although no specific nutritional status assessment tool has been recommended for patients with MG, the European Society for Clinical Nutrition and Metabolism’s malnutrition assessment criteria can be used (27). This assessment first involves screening for malnutrition using tools such as the Nutrition Risk Screening 2002 (28), the Mini Nutritional Assessment (29), and the Malnutrition Universal Screening Tool (30). Diagnosis and grading can then be conducted based on criteria including weight loss, muscle mass, and food intake. Although ESPEN guidelines recognize that patients with Myasthenia Gravis may suffer from dysphagia, they do not provide disease-specific nutritional recommendations for MG. Thus, current clinical guidance on nutritional management remains generalized and highlights the need for further research in this area.

Given the role of inflammation in MG pathogenesis, nutritional interventions have been explored as a potential strategy for disease management (Tahir and Ashraf). In studies of individuals with autoimmune diseases that share a similar pathogenesis, it has been reported that a Western-style diet, characterized by a high intake of saturated fats and animal-based foods, exacerbates the symptoms of multiple sclerosis (31) and rheumatoid arthritis (32). Additionally, it has been noted that foods rich in saturated fat content trigger the production of the pro-inflammatory cytokines Th1 and Th17 (33), whereas polyunsaturated fatty acids significantly reduce inflammation through the production of prostaglandins E1 and E2. A diet high in vegetables, fruits, and fiber can improve autoimmune disease flare-ups by regulating the function of short-chain fatty acids and regulatory T cells in the intestinal barrier (34).

Because MG directly affects muscle function, adequate protein intake is important to prevent muscle loss. In studies conducted, low serum albumin levels have been found to be associated with disease severity in patients with MG. As albumin levels decreased, it was observed that MG symptoms became more severe. Additionally, a significant relationship was found between low albumin levels and complications such as length of hospital stay and respiratory failure. Nutrition and anti-inflammatory dietary strategies can play an important role in disease management; therefore, these patients should be regularly monitored and necessary interventions should be made (35).

### 2.1 Myasthenia gravis and microbiota

The relationship between nutrition and MG appears to be a bidirectional one. Gut microbiota influence immune system activation, and alterations in microbiota composition (i.e., dysbiosis) have been reported in various autoimmune diseases, including MG. While some evidence suggests that dysbiosis may contribute to proinflammatory responses associated with MG progression, the direction and strength of this relationship remain unclear (36). Some studies have reported an increased prevalence of MG in individuals with inflammatory bowel disease (37). Additionally, Su et al. (38)explored a potential causal link and hypothesized that microorganisms with pro-inflammatory characteristics could increase susceptibility to MG. However, further causal inference studies are required to validate this association. Alterations in the diversity and composition of gut microbiota have also been observed in MG patients, suggesting that the disease itself may influence microbiota dynamics. For instance, Moris et al. (39) reported that patients with MG exhibited reduced levels of verrucomicrobia, actinobacteria, and bifidobacterium, alongside increased levels of bacteroides and desulfovibrio, compared to healthy controls. These microbial shifts were associated with increased intestinal permeability and immune dysregulation—namely, an imbalance between FoxP3 + CD4 + regulatory T cells and Th17 cells potentially exacerbating disease severity. Nonetheless, these findings should be interpreted as preliminary, and more mechanistic and longitudinal studies are needed to determine causality and clinical relevance (39).

Meanwhile, Rinaldi et al. (40) injected bifidobacterium into mice with MG to evaluate its therapeutic effects, examining neuromuscular symptoms, inflammation and damage degree, cytokine profiles, and immune cell activities. They noted an improvement in symptoms, a decrease in antibody levels and T cell activity, and an increase in anti-inflammatory markers TGFβ and FoxP3.

An increase in the number of circulating regulatory T cells can enhance autoimmune responses in MG. This suggests that MG can be affected by factors like hormone therapy, diet, or microbiota composition like other autoimmune diseases (41). Therefore, it is likely that the symptoms of the disease can be reduced by ensuring healthy microbiota in the intestines. Some sources recommend following the Mediterranean diet model to manage MG (17).

### 2.2 Diet and nutritional models

In the treatment of MG, the potential of nutritional strategies for successful disease management is gaining increasing importance. The anti-inflammatory and immunomodulatory effects of certain dietary patterns and nutrients are noteworthy in terms of improving MG symptoms and enhancing patients’ QoL. Studies evaluating the effects of different dietary models on MG are summarized in Table 1. However, these are mostly small-scale and preliminary studies, and there is a clear need for high-quality, large-scale clinical trials to confirm their findings and translate them into clinical practice.

**Table 1:** Prior studies on dietary treatments for MG.

Study	Type	Diet	Mechanism	Results	Level of evidence*	Comment
(73)	CS	Whole plant-based diet	Inflammation and oxidative stress reduction	Improvement in muscle strength; symptom reduction	4	Reported symptom improvement; limited by single-patient design.
(48)	CS	Intermittent fasting during Ramadan	Otofagia; immune system regulation	Stable symptoms and clinical course for patients with mild MG only	2b	Shows an association with clinical outcomes; no causal inference.
(56)	CS	Complete exclusion of gluten	Gut-immunity interaction; inflammation reduction	Symptom relief; regulation of neuroimmune responses	5	Very limited evidence; rare and anecdotal associations with MG.
(51)	RCT (ongoing)	High-fat, low-carbohydrate, moderate-protein diet	Metabolism regulation; improvement of mitochondrial functions; inflammation reduction	Reduction in clinical symptoms; improvement in muscle strength	(Pending) → likely 1b	Potentially high-quality evidence; results pending.
(40)	AE	Bifidobacterium supplementation	Reduction of proinflammatory cytokines; increase in anti-inflammatory responses; suppression of autoimmune responses	Reduction in inflammation; improvement in symptoms	5	Experimental evidence only; useful for hypothesis generation.
(61)	CS	Vitamin D supplementation	Immune system regulation; inflammation reduction; neuromuscular transmission support	Reduction in MG symptoms	4	Specific clinical context (post-gastrectomy); limited generalizability.

CS, case study; RCT, randomized control trial; AE, animal experiment.

*According to Oxford Centre for Evidence-Based Medicine 2011 (OCEBM) (74).

#### 2.2.1 Plant-based diets

With a vegan diet entirely of plant-based foods, immunity can be supported by consuming low amounts of saturated fats and cholesterol while getting high amounts of vitamins A and C, fiber, and phytochemicals (42). Plant-based antioxidants exhibit anti-inflammatory effects by regulating free radicals that cause oxidative stress and pro-inflammatory cytokines, which play a role in the pathogenesis of autoimmune diseases (43). Additionally, the consumption of whole grains, fruits, and vegetables increases the production of short-chain fatty acids in the intestines and strengthens the body’s defense mechanism against pathogens (44).

Yiaslas et al. (45) found that a 65-year-old male patient with multiple morbidities, including myasthenia gravis, experienced significant clinical improvements after adopting a whole food, plant-based diet for 5 months. His systolic blood pressure decreased by 15.1% (from 126 mmHg to 107 mmHg), fasting glucose dropped by 19.5% (from 113 mg/dL to 91 mg/dL), and he lost approximately 19.3% of his body weight (from 255 lbs. to 205.7 lbs). Additionally, his LDL cholesterol was reduced by 42% (from 38 mg/dL to 22 mg/dL within 1 month), and he reported complete elimination of angina symptoms. These outcomes indicate not only cardiometabolic improvement but also potential alleviation of neuromuscular symptoms associated with MG.

#### 2.2.2 Intermittent fasting

Intermittent fasting has been proposed to improve metabolic health more than classical calorie restriction models (46). There are many different intermittent fasting models available featuring various hunger-satiety cycles, meal timing, and energy intake. The alternate fasting diet (5:2) is a dietary model in which less than 25% of the weekly energy requirement is consumed on 2 days a week, with no restrictions on the other days. However, in time-restricted dieting, food intake is limited to a maximum of 8 h each day. In a periodic fasting diet, fasting in time-restricted dieting, food intake is limited to a maximum of 8 h each day. In a periodic fasting diet, fasting for up to 24 h is done once or twice a week, with normal food intake on the other days (47).

In a study conducted on 108 patients to evaluate the effects of fasting during Ramadan on MG symptoms, Ismail et al. (48) reported that some experienced a worsening of symptoms during fasting, while others showed no change. Specifically, 71.3% of the patients (77 individuals) reported no change in their symptoms, 17.6% (19 individuals) experienced worsening, and 11.1% (12 individuals) reported improvement during the fasting period. This situation was primarily observed in patients with more severe symptoms who required regular medication. The study concluded that patients with mild MG could fast from food with appropriate medication adjustments under medical supervision, but that hydration was necessary. Ramadan fasting, unlike intermittent fasting, involves not consuming any liquids (including water) during daylight hours. More studies are necessary to evaluate the effects of intermittent fasting on MG.

#### 2.2.3 Ketogenic diets

A ketogenic diet is characterized by the daily consumption of less than 50 grams of carbohydrates and a high amount of fat. The resultant decrease in insulin secretion and depletion of glycogen stores due to low carbohydrate intake stimulate gluconeogenesis and ketogenesis (49). It is thought that this change in the metabolic process and the increased ketone bodies can lead to weight loss, reduce inflammation and enhance antioxidant capacity. Prior studies have evaluated the effectiveness of the ketogenic diet in treating epilepsy, obesity, and many neurodegenerative diseases (50). In a recent study by Stascheit and Makus (51), patients who followed a ketogenic diet for 12 weeks displayed an increase in MG-specific QoL and T cell activation, a reduction in the severity of their symptoms, and an improvement in energy compared with the group that did not follow the diet. However, the definitive results of the study have not yet been published.

#### 2.2.4 The Mediterranean diet

The Mediterranean diet, which is based on the consumption of vegetables and fruits, whole grains, olive oil, fish, and nuts and is a model of healthy eating, contains low levels of saturated fats, sugars, and processed foods, as well as high amounts of antioxidants and polyphenols. Thanks to its healthy content, it supports cardiovascular health and exhibits anti-inflammatory properties (52).

Although there is still no direct study examining its effect on MG, the Mediterranean diet has been recommended for this patient group due to its anti-inflammatory effects (17). Some scientists have also found that the Mediterranean diet exhibits a neuroprotective effect by suppressing neuroinflammatory gene expressions and reducing inflammation in the central nervous system (53). In addition, they demonstrated that dietary intervention suppresses systemic inflammation by reducing inflammation markers in the peripheral blood circulation. The Mediterranean diet likely has the potential to reduce inflammation in MG as well, which would lead to an alleviation of its symptoms.

#### 2.2.5 The gluten-free diet

The activation of the immune system in an autoimmune disease increases the risk of developing another autoimmune disease (54). Barley, wheat, and rye all contain gluten, which can be associated with other autoimmune diseases in addition to celiac disease through its potential for immune system activation (55). Gluten also increases intestinal permeability, allowing bacteria and toxic metabolites to reach the central nervous system through the bloodstream. The disruption of blood–brain barrier integrity then activates microglial cells and triggers neuronal inflammation. These cumulative changes thus lead to the development of neurodegenerative diseases through the disruption of the microbiota–gut–brain axis (47).

A study found that a 34-year-old woman with celiac disease and MG showed improvement in the gastrointestinal symptoms of both diseases after starting a gluten-free diet. After 2 months on the diet, the patient reported the complete resolution of diarrhea and abdominal pain, and a marked reduction in generalized muscle weakness and fatigue. Laboratory testing also showed normalized anti-tissue transglutaminase IgA antibody levels, supporting dietary adherence and response (56). However, no definitive conclusion has been reached regarding whether gluten alone can affect MG symptoms. Considering the connection between the gut and the brain, it is likely that neurological symptoms will also improve along with gastrointestinal symptoms.

#### 2.2.6 Vitamin and mineral supplementation

Vitamin D regulates T cells by suppressing the production of inflammatory cytokines, demonstrating a strong immunomodulatory effect (57). Vitamin D deficiency has been associated with chronic autoimmune diseases like multiple sclerosis (58), systemic lupus erythematosus (59), and rheumatoid arthritis (60). In patients with MG, plasma 25(OH)D levels have also been found to be lower compared to healthy individuals. In a case study examining a 62-year-old male patient with low vitamin D levels, vitamin D supplementation resulted in significant improvements in both MG symptoms and overall muscle function. Specifically, the patient’s serum 25(OH)D level increased from 6.64 ng/mL to 39.3 ng/mL over 3 months, and the Absolute and Relative Score of MG (ARS-MG) decreased from 18 to 2. Additionally, the MG-Activities of Daily Living (MG-ADL) score improved from 18 to 1 during the same period, indicating substantial enhancement in muscle strength and daily functioning (61).

Because different inflammatory or proinflammatory mediators are involved in the pathogenesis of MG, targeting inflammation in treatment has been suggested as an important strategy in disease management (62). Therefore, it is important to provide nutrients help prevent or manage of inflammation. For this purpose, in addition to vitamin D, the intake of vitamins A and E, zinc, polyphenols, and polyunsaturated fats should be increased, and the consumption of foods high in simple sugars, salt, and saturated fats should be limited (63, 64). However, studies directly examining the effects of these nutrients on MG are quite limited. There is therefore a need for more extensive and high-quality research in this area.

## 3 Nutritional recommendations for myasthenia gravis treatments

### 3.1 Medications

#### 3.1.1 Acetylcholinesterases

The main component of pyridostigmine, commercially available as Mestinon, inhibits the enzyme acetylcholinesterase, allowing acetylcholine to remain longer at the nerve-muscle junction and temporarily improving nerve-muscle transmission. This mechanism helps reduce muscle weakness in patients with MG. While side effects can include increased salivation and bowel movements, nausea, vomiting, muscle cramps and hypotension, these are generally mild and manageable (65). In case of diarrhea, foods with high fat, spices, and fiber content should be avoided. Instead, foods that are easy to digest, relax the intestine, and provide fluid-electrolyte balance are preferred (66).

#### 3.1.2 Immunosuppressive drugs

Corticosteroids are another group of drugs used in MG treatment. These drugs have a strong immunosuppressive and anti-inflammatory effect, reducing the production of antibodies against acetylcholine receptors, which is the main problem in MG. This improves overall neuromuscular transmission and reduces muscle weakness (67). In addition, immunosuppressive drugs containing azathioprine, methotrexate, and mycophenolate mofetil suppress the immune response by affecting the activation of T and B cells (68).

However, corticosteroids have many side effects. These include unintentional weight gain, glucose intolerance, osteoporosis, high blood pressure, sleep disturbances, and psychological problems (69). Various dietary recommendations have been developed to minimize these side effects, with a focus on the intake of calcium, Vitamin D, protein, fiber, and fluids (70).

### 3.2 Thymectomy

The thymus gland plays a critical role in the development and regulation of the immune system. After puberty, the thymus gland usually begins to shrink and disappears almost completely during adulthood. However, Tireli et al. (71) found that in patients with MG, this expected shrinkage does not occur; instead, pathological changes like the abnormal growth of the thymus gland (thymic hyperplasia) or tumors (thymoma) occur. Abnormalities of the thymus gland in patients with MG can contribute to their impaired autoimmune responses and muscle weakness. The presence of thymic hyperplasia or thymoma may further affect the severity of the disease and responses to treatment.

During treatment, the thymus gland may be removed to restore the immune system and reduce symptoms (72). However, in the post-operative period, 50% of patients are at risk of developing various complications that can increase morbidity and mortality. Immunonutritional products like glutamine, arginine, and w-3 can be used to support the immune system and make the treatment more effective. A study of 244 patients with MG reported that those who received preoperative immunonutrition had fewer complications and shorter hospital stays than those who received standard nutrition (64), demonstrating the continued importance of nutrition even in the presence of other treatments.

## 4 Conclusion

Although immunosuppressive drugs and other methods are used to treat MG, nutritional status plays an important role in its management. Dysphagia and malnutrition are common in patients and can have a significant negative impact on both QoL and treatment outcomes. Emerging evidence suggests that anti-inflammatory dietary strategies such as increasing the intake of vegetables, fruits, and fiber-rich foods while reducing saturated fats and animal products may help alleviate symptom severity by modulating systemic inflammation and gut microbiota. Moreover, ensuring adequate protein intake appears to support muscle mass preservation, which is essential in a disease characterized by muscle weakness.

Therefore, routine nutritional assessment and individualized dietary interventions should be considered an integral part of MG care. While further controlled studies are needed, current findings underscore the clinical relevance of incorporating dietitians into multidisciplinary teams managing MG, especially in cases complicated by dysphagia or malnutrition. Nutritional support should not be viewed merely as an adjunct but as a potentially impactful element in optimizing clinical outcomes for individuals with MG.
